# RETRACTION: Combined Anticancer Effects of Simvastatin and Arsenic Trioxide on Prostate Cancer Cell Lines via Downregulation of the VEGF and OPN Isoforms Genes

**DOI:** 10.1111/jcmm.71059

**Published:** 2026-02-12

## Abstract

**RETRACTION**: MirzaeiA. , RashediS. , AkbariM. R. , KhatamiF. , and AghamirS. M. K. , “Combined Anticancer Effects of Simvastatin and Arsenic Trioxide on Prostate Cancer Cell Lines via Downregulation of the VEGF and OPN Isoforms Genes,” Journal of Cellular and Molecular Medicine 26, no. 9 (2022): 2728‐2740, 10.1111/jcmm.17286.35366048 PMC9077302

The above article, published online on 02 April 2022 in Wiley Online Library (wileyonlinelibrary.com), has been retracted by agreement between the authors; the journal Editor‐in‐Chief, Stefan N. Constantinescu; the Foundation for Cellular and Molecular Medicine, and John Wiley & Sons Ltd. The retraction has been agreed upon following an investigation into concerns raised by a third party. The investigation identified duplication of elements between Figures 3 and 4 in this article. Additional duplications were found between Figures 1, 3, and 4 of this article and figures in other articles published by some of the same authors. Not all authors of the article were involved in the later publications where the additional duplicated elements were found. Although those other articles were published later, some of the duplicated elements were used to represent different scientific contexts. The authors cooperated with the investigation and explained that these duplications were inadvertent errors resulting from figure mismanagement. However, they were unable to provide their original raw data. Due to the extent and nature of the concerns, the editors consider the results and conclusions to be unreliable.

**RETRACTION**: A. Mirzaei , S. Rashedi , M. R. Akbari , F. Khatami , and S. M. K. Aghamir , “Combined Anticancer Effects of Simvastatin and Arsenic Trioxide on Prostate Cancer Cell Lines via Downregulation of the VEGF and OPN Isoforms Genes,” Journal of Cellular and Molecular Medicine 26, no. 9 (2022): 2728‐2740, 10.1111/jcmm.17286.35366048 PMC9077302

The above article, published online on 02 April 2022 in Wiley Online Library (wileyonlinelibrary.com), has been retracted by agreement between the authors; the journal Editor‐in‐Chief, Stefan N. Constantinescu; the Foundation for Cellular and Molecular Medicine, and John Wiley & Sons Ltd. The retraction has been agreed upon following an investigation into concerns raised by a third party. The investigation identified duplication of elements between Figures 3 and 4 in this article. Additional duplications were found between Figures 1, 3, and 4 of this article and figures in other articles published by some of the same authors. Not all authors of the article were involved in the later publications where the additional duplicated elements were found. Although those other articles were published later, some of the duplicated elements were used to represent different scientific contexts. The authors cooperated with the investigation and explained that these duplications were inadvertent errors resulting from figure mismanagement. However, they were unable to provide their original raw data. Due to the extent and nature of the concerns, the editors consider the results and conclusions to be unreliable.

